# Addressing COVID-19 inequities using bidirectional crisis and emergency risk communication and vaccine clinic interventions: a descriptive study

**DOI:** 10.1186/s12889-023-16410-3

**Published:** 2023-08-10

**Authors:** Abby M. Lohr, Kelao Charmaine Neumbo, Jane W. Njeru, Luz Molina, Rachel Hasley, Yahye Ahmed, Onelis Quirindongo-Cedeno, Gloria A. Torres-Herbeck, Miriam L. Goodson, Ahmed Osman, Jenny A. Weis, Mark L. Wieland, Irene G. Sia

**Affiliations:** 1 Division of Community Internal Medicine, Geriatrics, and Palliative Care, Mayo Clinic, Rochester, USA; 2 Case Western Reserve University, Cleveland, USA; 3 Division of Public Health, Infectious Diseases and Occupational Medicine, Mayo Clinic, Rochester, USA; 4 Community Based Research, Mayo Clinic, Rochester, USA; 5 Divanyx, Rochester, USA; 6 Alliance of Chicanos, Hispanics, and Latin Americans, Rochester, USA; 7 Intercultural Mutual Assistance Association, Rochester, USA; 8 Research Administrative Services, Mayo Clinic, Rochester, USA

**Keywords:** COVID-19, Health equity, Immigrants, Refugees, Community-based participatory research, Vaccination, Health Care Quality, Access, Evaluation

## Abstract

**Background:**

Im/migrants (immigrants and migrants, including refugees, asylum seekers, and individuals without legal documentation) experience unique assets and needs in relation to coronavirus disease 2019 (COVID-19). Community-based participatory research (CBPR) is one way to engage im/migrant communities. Rochester Healthy Community Partnership (RHCP) is a CBPR partnership in Rochester, Minnesota. RHCP partners noted that credible COVID-19 information was not available to their communities. In response, RHCP formed a COVID-19 Task Force and adapted the Centers for Disease Control and Prevention’s Crisis and Emergency Risk Communication (CERC) framework to create an intervention that prioritized im/migrant groups experiencing health disparities. In the CERC intervention, communication leaders delivered COVID-19 health messages to their social networks and documented related concerns. RHCP relayed these concerns to regional leaders to ensure that im/migrant experiences were included in decision making. Once vaccines were available, RHCP continued to deploy the CERC intervention to promote vaccination equity. The aims of this paper are to (1) describe the implementation of a bidirectional CERC intervention for vaccination equity, and (2) describe a community-engaged and community-based vaccine clinic intervention.

**Methods:**

First, we surveyed participants (*n* = 37) to assess COVID-19 experiences, acceptability of the CERC intervention, and motivation to receive a COVID-19 vaccination. Second, we collaborated with community partners to hold vaccine clinics. We report descriptive statistics from each intervention.

**Results:**

When asked about the acceptability of the CERC intervention for vaccine equity, most participants either reported that they ‘really liked it’ or ‘thought it was just ok’. Most participants stated that they would recommend the program to family or friends who have not yet received the COVID-19 vaccine. Almost all participants reported that they felt ‘much more’ or ‘somewhat more’ motivated to receive a COVID-19 vaccine after the intervention. We administered 1158 vaccines at the vaccination clinics.

**Conclusions:**

We found that participants viewed the CERC intervention for vaccination equity as an acceptable way to disseminate COVID-19-related information. Nearly all participants reported that the intervention convinced them to receive a COVID-19 vaccine. In our experience, community-engaged and community-based clinics are a successful way to administer vaccines to im/migrant communities during a pandemic.

**Supplementary Information:**

The online version contains supplementary material available at 10.1186/s12889-023-16410-3.

## Background

The coronavirus disease 2019 (COVID-19) pandemic has had a devastating impact on all our lives. Yet, due to racism and its economic and social consequences in the United States (US), some populations have been more impacted than others including Non-Hispanic Black and Hispanic communities [1–5]. Despite only representing 18% of the US population, Hispanics made up 25% of all cases of COVID-19 as of March 9, 2022 [6]. Black and Hispanic populations also have a higher risk for hospitalization compared to their non-Hispanic White counterparts. For example, in a systematic review examining racial and ethnic disparities in COVID-19 outcomes, Mackey et. al. found seven studies suggesting that Black populations are 1.5 to 3 times more likely to be hospitalized because of COVID-19 infection compared with White populations. Concurrently, these authors identified two studies with findings suggesting that Hispanic populations have a 1.5 times higher risk of hospitalization compared to non-Hispanic Whites [3]. In New York City—where COVID-19 took a heavy toll on residents—in May 2020 there was a two-fold higher age-adjusted death rate for the Hispanic compared to the non-Hispanic White population (204.6/100,000 vs. 101.3/100,000 people) [7]. Similarly, the Centers for Disease Control and Prevention (CDC) reported that Black or African American, non-Hispanic persons are 1.7 times more likely to die from COVID-19 compared to their White, non-Hispanic counterparts [8].

These disparities extend to COVID-19 vaccinations, due to hesitancy and access. In a literature review combining 13 studies with over 100,000 participants conducted in early 2021, Khubchandani and Macias found that the overall COVID-19 vaccination hesitancy for adults in the US was 26% but was much higher for Black (42%) and Hispanic (30%) populations. The major predictors of vaccine hesitancy among Blacks and Hispanics included younger age, identifying as female, low income or formal education level, larger household size, mistrust of the medical system and history of racial discrimination as well as greater exposure to misinformation, perceived risk of COVID-19 infection, past vaccine-related behaviors and beliefs, and concerns about the safety and efficacy of the COVID-19 vaccine [9]. Similarly, in a survey of over 200 Black Americans in 2020, investigators found high levels of vaccine hesitancy. The participants attributed their mistrust to systemic racism—including discrimination and mistreatment by the healthcare system and the government [10]. Even when Black or Hispanic individuals choose to receive a COVID-19 vaccination, they often experience disparities in access due to unequal distribution of vaccine doses, inaccessible locations, inconvenient times, and underinvestment in healthcare services in Black, Indigenous, and People of Color (BIPOC) communities [11].

Although often documented as part of larger racial and ethnic groups and rarely prioritized as their own population, im/migrants (immigrants and migrants, including refugees, asylum seekers, and individuals without legal documentation) in the US experience unique needs related to COVID-19. Im/migrants experience numerous barriers to vaccination such as lack of COVID-19 information or misinformation [12], language barriers, challenges reaching vaccination sites [12, 13], the inability to take time off work or stay home if they experienced side effects [12], as well as lack of trust in a healthcare system run by predominately White individuals, challenges in healthcare access [14], working in front-line positions, and living in high density housing [15].

Immigration status can also influence vaccine acceptance. Im/migrants without legal documentation may be reluctant to receive a COVID-19 vaccine because they fear encountering immigration authorities in the process [16]. Additionally, the Trump Administration’s redefinition of ‘public charge’ (which certified that using public assistance services could be cause for rejection of residency) resulted in some im/migrants worrying that receiving a free vaccine would be included in this definition [17]. To address these COVID-19 disparities, Somali community members in Minnesota (MN) suggest that we must engage im/migrant communities, ensure that community members feel that their voices are heard, facilitate relationship building between im/migrants and representatives from healthcare and government agencies, and include im/migrants in decision making [18]. To build trust, we must also address communication barriers and experiences of racism [18].

One potential way to engage im/migrant communities and follow these suggestions is by using a community-based participatory research (CBPR) approach [19]. CBPR “is a collaborative approach to research that equitably involves, for example, community members, organizational representatives, and researchers in all aspects of the research process” [20]. Rochester Healthy Community Partnership (RHCP) is a CBPR partnership in Rochester, MN which has successfully deployed evidence-based, community-engaged, National Institutes of Health-funded research projects in the areas of tuberculosis, Type 2 diabetes, and chronic disease prevention [21–23] (https://rochesterhealthy.org/). We primarily partner with im/migrant populations. Thus, with well-established community-academic partnerships, RHCP (hereafter also referred to as ‘we’) was well positioned to address COVID-19 related disparities in southeastern MN.

At the beginning of the pandemic, RHCP im/migrant partners noted that credible COVID-19 information was not readily available to their communities. This experience was not unique and has been noted among other im/migrant populations around the world [24, 25]. In response, RHCP formed a community-based COVID-19 Task Force and adopted the CDC’s Crisis and Emergency Risk Communication (CERC) framework [26] to create a bidirectional intervention that prioritized im/migrant groups in Olmsted County experiencing health disparities. We have previously described our mixed methods evaluation of the CERC intervention to address COVID-19 prevention, testing, and socioeconomic impacts [27, 28]. Here we explain our adaptation of this intervention to address vaccination equity.

### Theoretical framework

We applied Rothman’s community intervention approaches [29]. This community organization model includes three strategies: (1) planning/policy (using data to propose and enact solutions), (2) community capacity development (assuming “that change is best accomplished when the people affected by the problems are empowered with the knowledge and skills needed to understand their problems, and then work cooperatively together to overcome them”), and (3) social advocacy (applying pressure to people or institutions that caused or are maintaining a social inequity) [29]. In our case, RHCP developed credible COVID-19 information in numerous languages and formats disseminated through various media and implemented accessible vaccination clinics that prioritized the im/migrant community [27, 28]. In Rothman’s model, this work is characterized as a mix between strategies one and two: planning/policy with substantial capacity development. Together these strategies are called participatory planning. A key tenet of participatory planning is collaborating with community-based groups to incorporate all perspectives when defining goals. Community members may then have a vested interest in implementing the plans they created [29].

### Objective

The aims of this paper are twofold. First, we describe the implementation of the bidirectional CERC intervention for vaccination equity. Second, we describe a community-engaged and community-based vaccine clinic intervention. We used the template for intervention description and replication (TIDieR) checklist to guide our reporting [30].

## Methods

### Setting and participants

Olmsted County is in Southeast MN and has a population of 163,000 people [31]. Rochester is the largest city with a population of 121,000 people [32]. By November 19, 2020, 5,889 patients had tested positive for COVID-19 in Olmsted County, 30% of positive tests were among Black and Hispanic groups, despite representing only 16% of the population.

### Interventions

#### Bidirectional crisis and emergency risk communication (CERC) intervention for vaccination equity

##### Recruitment

In the CERC intervention to address COVID-19 prevention, testing, and socioeconomic impacts, RHCP community partners recruited communication leaders (CLs) based on their trustworthiness, credibility in the community, and authenticity of social network engagement. The CL eligibility criteria were self-identification as a leader within a social network and age 18 or older. The CL’s role included delivering messages to their social networks as well as documenting and sharing COVID-19-related concerns and impacts. RHCP then relayed that information to regional leaders to ensure that im/migrant experiences were included in decision making. We recruited 24 CLs in March 2020, who then reached more than 39,000 individuals in the first 9 months of the RHCP CERC intervention to address COVID-19 prevention, testing, and socioeconomic impacts [27].

##### Evaluation

Beginning in January 2021, a subset of four CLs and their social networks participated in an evaluation of the bidirectional CERC intervention for vaccination equity. Based on county demographics, RHCP participation, willingness to participate, and disproportionate COVID-19 impact, we limited CL participation to those who self-identified as Black or African American (*n* = 2) or Hispanic/Latino (*n* = 2) im/migrants. Both Black CLs were first or second-generation Somali im/migrants. While each CL’s social network may have included individuals from outside their racial/ethnic group, inclusion criteria for CL network members to participate in measurements included self-identification as Black or Hispanic/Latino im/migrants.

##### Message adaptation

Within the COVID-19 Task Force, RHCP formed a communication working group which used a seven-step process to adapt and distribute COVID-19 messaging.

1. The communication working group first developed message maps: an organized process of displaying detailed responses to anticipated questions or concerns [33]. The CL’s feedback guided us in anticipating questions. The message maps focused on vaccine efficacy, side effects, misinformation, and local details on how to access vaccines.
2. To address each topic, the communication working group searched for COVID-19 messages from credible sources (e.g., the websites of the Minnesota Department of Health, Olmsted County Public Health, the CDC, the World Health Organization, and Mayo Clinic). Communication working group members used these sites to a) identify appropriate answers to community questions, usually raised by the CLs during meetings, b) find new COVID-19 vaccine information, and c) identify resources for COVID-19 vaccination in the community.
3. The communication working group adapted the messages to make them culturally appropriate (e.g., by including images of Somali or Hispanic/Latino individuals) and added information about pertinent, local social and economic health resources.
4. The communication working group chose the most appropriate message format (video, infographic, or animated graphic) and prepared a draft in English.
5. The RHCP COVID-19 Task Force reviewed the draft to ensure cultural appropriateness, accurate response to community questions, and consensus on dissemination method.
6. The communication working group incorporated feedback from the task force, finalized the message, and collaborated with the Mayo Clinic Language Department or other community-based services to translate the message into Spanish and Somali. Of note, other ethnic groups also adopted the messages into additional languages (e.g., Anuak which is spoken in western Ethiopia and South Sudan), but our evaluation focused on the two largest groups: Spanish and Somali speakers.
7. The communication working group distributed the final messages to the CLs via e-mail, WhatsApp, text message, or Facebook messenger and to the larger community through the RHCP Facebook page and website (see example in Additional file 1 and message library at www.rochesterhealthy.org/covid-19).

##### Intervention design

Because of physical distancing guidelines, CLs delivered the adapted messages virtually: through social media platforms. In our pilot study, we found that the type of social media platform was not important to the reach or acceptability of the intervention. Accordingly, each CL chose the platform (e.g., Facebook, WhatsApp, group text chat) most accessible and widely used by their network members with the caveat that the platform must support dissemination of videos and infographics. We kept the groups closed to minimize contamination from individuals outside the study. We previously reported on message reach and engagement [27].

CLs participated in individualized training via a one-hour video conference before beginning the intervention. After training, each week CLs used one social media platform to distribute two vaccine-related COVID-19 messages to their social networks, respond to questions, and solicit social and economic concerns. Bilingual RHCP staff systematically tracked these concerns and highlighted them during RHCP COVID-19 Task Force discussions.

CLs, RHCP community and academic partners, and county public health department staff attended weekly 60-min, virtual meetings throughout the intervention interval for three purposes. First, CLs shared their progress and taught emerging best practices. Second, to ensure we disseminated the most accurate and up to date information, RHCP refined existing messages and generated new messages in response to community feedback and rapidly changing COVID-19 facts and resources. Third, infectious disease experts (academic partners) or social or economic resource experts (community partners) answered questions in real time to clarify any misunderstandings or add greater detail.

**Fig. 1:**
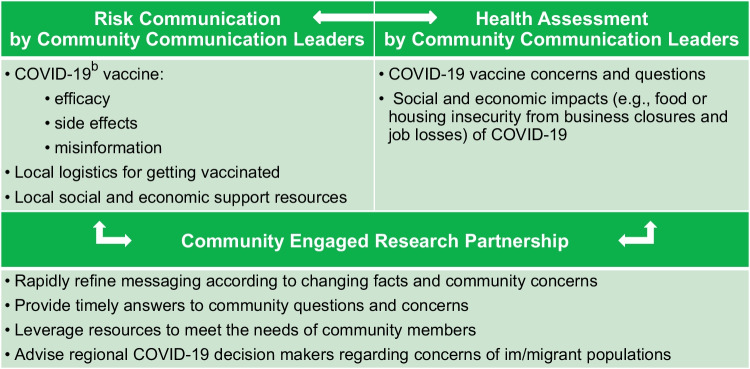
Rochester Healthy Community Partnership’s bidirectional crisis and emergency risk communication (CERC) intervention for vaccination equity^a^ ^a^ A version of this figure was published elsewhere: Wieland ML, Asiedu GB, Njeru JW, Weis JA, Lantz K, Abbenyi A. et al. Public Health Reports (Vol 137, Issue 2) pp. 352-361, copyright © 2022 SAGE Publications. Reprinted by Permission of SAGE Publications. ^b^ COVID-19 = coronavirus disease 2019

The bidirectional communication between community and academic partners was the most important facet of the intervention. We used feedback from community members to inform regional decision makers so that COVID-19 policies included im/migrant priorities. For example, local healthcare institutions addressed barriers to COVID-19 vaccination—including language non-congruence and challenges with scheduling platforms—through dialogue between the RHCP COVID-19 Task Force and clinical leaders (Fig. 1).

##### Intervention assessment (data collection)

We conducted a cross-sectional survey to assess knowledge and attitudes about COVID-19 vaccines, including intention to be vaccinated. We administered surveys to the four selected CLs and a subset of their social network members who were willing to participate in our evaluation (4 CLs + 33 network members = 37 participants). The number of participants surveyed does not represent the depth of the four participating social networks. Bilingual study staff (not CLs) used an institutional web-based data collection platform (REDCap) [34, 35] to obtain oral informed consent and administer surveys in-person following COVID-19 masking protocols. RHCP provided remuneration for study participation. To assess the bidirectional CERC intervention for vaccination equity, the same participants completed surveys after the intervention. The surveys included demographic, COVID-19-related, and intervention acceptability questions.

##### Demographic

Study participants reported the following demographic data: sex, age, ethnicity, country of birth, annual household income, education level, employment status, time lived in the US, and primary language spoken at home. We also included a question around English language proficiency adapted from the US Census Bureau [36].

##### COVID-19 survey measures

We assessed COVID-19 personal experiences around infection and death using questions adapted from the COVID-19 Community Response Survey [37]. Additionally, we examined how easy or difficult it was to understand and apply COVID-19 information using questions adapted from the European Health Literacy Survey Questionnaire [38]. Finally, we assessed COVID-19 perceived infection risk and preventive measures using items from the World Health Organization’s Survey Tool and Guidance for behavioral insights on COVID-19 [39].

##### Vaccination *CERC* intervention acceptability

Using questions adapted from “Making Health Communication Programs Work” from the National Cancer Institute, we asked participants about the CERC intervention acceptability, whether they would recommend it to others, and whether the intervention motivated them to receive a COVID-19 vaccine [40].

#### COVID-19 vaccine clinics

During the initial COVID-19 vaccine roll out, it became clear that a subset of im/migrant community members did not feel comfortable receiving their vaccine at healthcare institutions or pharmacies. To fill this gap, RHCP collaborated with multiple partners to address the unique needs around providing COVID-19 vaccinations to im/migrants in Olmsted County. Considerations included flexibility in time, ease of registration, location, and language translation. Although we did accept walk-ins, we used a pre-registration process to facilitate smoother clinic operations and vaccine allocation stewardship at a time when there was limited vaccine supply. RHCP contacted the Mayo Clinic COVID-19 Vaccine Allocation and Distribution Workgroup (COVAD) to propose holding community-based vaccine clinics. The Mayo Clinic COVAD group agreed and provided the vaccine as well as infrastructure around language interpretation and vaccine administration (nursing staff, vaccine education, and documentation of vaccination). RHCP staff and CLs promoted the vaccine clinics (see Fig. 1), pre-registered community members, sent reminders, followed-up with community members with information on the location and time for the second dose (where indicated), and volunteered at the clinics. Following CDC and Mayo Clinic guidelines, initially we only offered vaccines to adults but later included adolescents and children as they became eligible.

Thus, through regular meetings and troubleshooting, we organically developed a vaccine clinic process that not only met the needs of the community but also complied with Mayo Clinic standard protocols. To make the clinics easily accessible for the community, we hosted them at three elementary schools and a community education center with large im/migrant student populations and one non-profit that provides support services for im/migrants.

##### Data collection

At the community-based vaccine clinic, we collected the following information from those who received vaccines: age, gender, race, and ethnicity. We collected this information when participants registered to receive a vaccine – either within two weeks of the clinic or the day of the event.

### Data analysis

In this paper, we present the descriptive statistics on participant demographics, COVID-19 experiences, acceptability of the bidirectional CERC intervention for vaccination equity, and motivation to receive a COVID-19 vaccination. We also report descriptive statistics on those who received vaccinations at the RHCP COVID-19 vaccine clinics.

## Results

### Bidirectional crisis and emergency risk communication (CERC) intervention for vaccination equity

The mean age among the survey participants was 43 years (Standard deviation [SD] = 10 years) and most of the participants identified as female (86%). Education was divided among participants with about a quarter (26%) receiving eight grades or less and about a third (31%) completing a college or graduate degree. The majority of participants’ average yearly family income was less than $29,000 (63%). Nearly the entire cohort was born outside the US (91%) and most spoke either Somali (49%) or Spanish (49%) at home.

Many participants reported a past COVID-19 infection among themselves (42%) and people in their immediate social environment (91%). Two thirds of respondents (67%) knew someone who died from COVID-19. Our results show disagreement around how difficult it was to locate and comprehend COVID-19-related information: nearly half of participants reported that it was ‘very or somewhat difficult’ to find (47%) and understand (41%) vs. more than half of participants reported it was ‘somewhat to very easy’ to find (53%) and understand (59%). In contrast, most participants (72%) reported that it was ‘extremely to somewhat difficult’ to avoid a COVID-19 infection. Most participants specified that, in the last week, they had washed their hands with soap and water for at least 20 s (92%), wore a mask in public (94%), and ensured physical distancing in public (83%). When asked whether a healthcare provider had recommended a COVID-19 vaccine to them, only 27% of survey respondents replied, ‘Yes’ while 70% replied ‘Do not know’ (Table 1).

**Table 1:** Participant Demographics and COVID-19 Experiences from the Bidirectional Crisis and Emergency Risk Communication (CERC) Intervention for Vaccination Equity (*N* = 37) ^a^

Characteristic	(*N* = 37)
**Age (years)**	
Mean (SD)	43 (10)
**Gender,** N (%)	
Male	5 (14)
Female	30 (86)
**How much schooling have you had?** N (%)	
8 grades or less	9 (26)
Some high school	5 (14)
High school graduate or GED	6 (17)
Some college or technical school	4 (11)
College or graduate degree	11 (31)
**What is your average yearly family income?** N (%)	
$0 to $29,999	20 (63)
$30,000 to $49,999	4 (13)
$50,000 and higher	8 (25)
**Born outside the United States,** N (%)	32 (91)
**What language do you most commonly speak at home?** N (%)	
English	1 (3)
Somali	18 (49)
Spanish	18 (49)
**Limited English proficiency,** N (%)^**b**^	22 (60)
**Race/ethnicity: Hispanic or Latino,** N (%)	
Yes	16 (43)
No	21 (57)
**Past COVID-19 infection,** N (%)^**c**^	15 (42)
**People in immediate social environment have been infected with COVID-19,** N (%)^**c**^	32 (91)
**Know someone who has died from COVID-19,** N (%) ^**c**^	24 (67)
**How easy or difficult is it to find the information you need related to COVID-19?** N (%)^**d**^	
Very to somewhat difficult	17 (47)
Somewhat to very easy	19 (53)
**How easy or difficult is it to understand information about COVID-19,** N (%)^**d**^	
Very to somewhat difficult	15 (41)
Somewhat to very easy	22 (59)
**Avoiding an infection with COVID-19 is…** N (%)^**e**^	
Extremely to somewhat difficult	26 (72)
Somewhat to extremely easy	10 (28)
**During last 7 days, frequently washed my hands with soap and water for at least 20 s,** N (%)^**e**^	
Not at all	0
Sometimes	3 (8)
Very often	33 (92)
**During the last 7 days, wore a mask in public,** N (%)^**e**^	
Not at all	1 (3)
Sometimes	1 (3)
Very often	33 (94)
**During the last 7 days, ensured physical distancing in public,** N (%)^**e**^	
Sometimes	6 (17)
Very often	30 (83)
**Has a healthcare provider recommended that you get a COVID-19 vaccine?** N (%)^**f**^	
Yes	10 (27)
No	1 (3)
Do not know	26 (70)

^a^The percent missingness was between 3 and 14%

^b^English proficiency was measured using the question ‘How well do you speak English?’ *Not at all; Not very well; Well; Very Well* adapted from the United States Census Bureau [36]. Participants who responded *Not at all* or *Not very well* were included in the limited English proficiency group

^c^COVID-19 personal experiences were measured using the following questions adapted from the COVID-19 Community Response Survey [37]: To your knowledge, are you, or have you been infected with COVID-19? *No; Yes* Do you know people in your immediate social environment (for example, family and friends) wo are or have been infected with COVID-19 (suspected or confirmed)? *No; Yes* If *Yes:* Do you know someone who died from COVID-19? *No; Yes*

^d^Understanding and applying COVID-19 information was measured with the following questions adapted from the European Health Literacy Survey Questionnaire [38]: How easy or difficult would you say it is to (1) Find the information you need related to COVID-19? and (2) Understand information about COVID-19? Responses options were on a 4-point Likert scale: *Very difficult; Somewhat difficult; Somewhat easy; Very easy*

^e^COVID-19 perceived infection risk and preventive measures were measured using the following questions from the World Health Organization’s Survey Tool and Guidance [39]: (1) Avoiding an infection with COVID-19 is *Extremely difficult; Somewhat difficult; Somewhat easy; Extremely easy;* (2) During the last 7 days, which of the following measures have you taken to prevent infection from COVID-19? A. Frequently washed my hands with soap and water for at least 20 s; B. Wore a mask in public; C. Ensured physical distancing in public *Not at all; Sometimes; Very Often*

^f^Rochester Healthy Community Partnership wrote the question ‘Has a healthcare provider recommended that you get a COVID-19 vaccine?’

When asked about the acceptability of the CERC intervention, most participants either reported that they really liked it (53%) or thought it was just ok (44%). Similarly, most participants stated that they would recommend the program to family or friends who have not yet received the COVID-19 vaccine (85%). After participating in the CERC intervention, almost all participants reported that they felt much more (55%) or somewhat more (32%) motivated to receive a COVID-19 vaccine (Table 2).

**Table 2:** Bidirectional Crisis and Emergency Risk Communication (CERC) Intervention for Vaccination Equity: Acceptability and Perceived Motivation to get a COVID-19 Vaccination ^a, b^

Question	Total (*N* = 37) N (%)
**General reaction to the Community-Engaged Bidirectional Pandemic CERC Intervention with Minority Populations**	
Really like it	17 (53)
Think it was just OK	14 (44)
Did not like it	0 (0)
Do not know/refused	1 (3)
**Would you recommend the program to family or friends who have not yet received the COVID-19 vaccine?**	
Yes, definitely	28 (85)
Maybe	2 (6)
No, definitely not	2 (6)
Do not know/refused	1 (3)
**After completing the program, do you feel more motivated to receive the COVID-19 vaccine?**	
Yes, much more motivated	17 (55)
Somewhat more motivated	10 (32)
No, not at all motivated	3 (10)
Do not know/refused	1 (3)

^a^The percent missingness was between 5 and 14%

^b^The questions in Table 2 are adapted from “Making Health Communication Programs Work” from the National Cancer Institute [40]

### COVID-19 vaccine clinics

Between March 27^th^ and December 11^th^, 2021, we held thirteen vaccine clinics during which 1158 vaccines were administered to 985 individuals (173 individuals received both the first and second dose at our clinics). Among the youth participants, the average age for child (5–11 years) vaccine recipients was 8 years (SD = 2 years) and 14 years (SD = 2 years) for adolescents (12-17 years). Most vaccine recipients were adults (74%) with an average age of 40 years (SD = 14 years). Most individuals vaccinated identified as White (49%) or Other (33%) while fewer identified as Black (16%) or Asian (5%). Additionally, most individuals vaccinated were Hispanic (62%) (Table 3). It is important to note that not all participants were im/migrants. We welcomed any individual who met vaccine eligibility criteria and we did not ask for identification.

**Table 3:** Demographics of Individuals Vaccinated at 13 Rochester Healthy Community Partnership Sponsored Vaccine Clinics from March 27 to December 11, 2021: Rochester, Minnesota ^a^

Characteristic	Number of Vaccines Administered (*N* = 1158)	Number of Individual Patients Served (*N* = 985)
**Age (years), N (%)**		
5–11	176 (15)	145 (15)
12–17	119 (10)	97 (10)
18 +	847 (73)	727 (74)
**Gender, N (%)**		
Female	527 (46)	449 (46)
Male	584 (50)	490 (50)
**Race,**^**b**^**N (%)**		
White	590 (51)	481 (49)
Other	401 (35)	326 (33)
Black	178 (15)	159 (16)
Asian	51 (4)	48 (5)
**Ethnicity, N (%)**		
Hispanic or Latino	752 (65)	614 (62)
Not Hispanic or Latino	388 (34)	354 (36)

^a^The percent missingness was between 1 and 4%

^b^Because respondents had the option to select more than one race, the totals do not sum to the number of vaccines administered or the patients served

## Discussion

In this paper, we described the implementation of a bidirectional CERC intervention for vaccination equity as well as community-engaged and community-based vaccine clinics. We presented the (1) descriptive statistics and results on acceptability from a survey measuring the impact of the CERC intervention and (2) descriptive statistics to demonstrate the results of a series of vaccine clinics developed in collaboration with im/migrant communities.

Using Rothman’s participatory planning strategy as a guide [29], as a partnership we first set a goal of improving access to COVID-19-related information and vaccines for im/migrants. We then centered community voices during CERC implementation by promoting active participation and empowerment of community members experiencing COVID-19-related health inequities. The survey results indicate that the bidirectional CERC intervention for vaccination equity is an acceptable way to disseminate COVID-19-related information to im/migrant communities. Nearly all respondents reported that participating in the intervention convinced them to receive a COVID-19 vaccine. Additionally, by holding vaccine clinics at community sites, iteratively creating a process that complied with Mayo Clinic protocols, and facilitating community member participation, we administered over 1100 vaccines. In our experience, using participatory planning to develop community-engaged and community-based clinics is a successful way to administer vaccines to im/migrant communities during a pandemic.

Similarly, in response to COVID-19-related disparities experienced by the Somali community, Reget et al. conducted a proactive, telephone-based intervention to call Somali patients over 65 years of age at one clinic in Minneapolis, MN. During the calls, bilingual volunteers provided COVID-19 education and ensured that patients were receiving continued care for non-COVID-19 related conditions. The investigators found that the calls were valued by the patients as a way to receive information and communicate with the clinic [41]. Likewise, after observing that public health information was not reaching Spanish and Arabic-speaking communities, Pereira, Naguib, and Siktberg partnered with the Nashville Metro Public Health Department as well as Hispanic and Egyptian community leaders in Tennessee to develop culturally sensitive, language congruent videos detailing COVID-19-related public health measures. The authors described the response as ‘overwhelming.’ After their videos spread widely over social media, the state invested more resources in the program and community members expressed gratitude for government support [42].

Our vaccination clinic experience is similar to work described by others. In Clarkston, Georgia, Malone et. al. used a culturally sensitive approach at a primary care clinic where providers partially or fully vaccinated 3127 individuals from January to May 2021 in a community that has served as a refugee resettlement site for 30 years. The authors attributed their success to established trusting relationships within the community, a user-friendly registration process, and consistent appointment times and location [43]. Marquez et al., developed a COVID-19 vaccination strategy that prioritized the Latinx population in San Francisco. This group vaccinated over 7,000 Latinx individuals, most of whom were first generation im/migrants. These authors attributed their success to the demand generated by trusted social network members who acted as messengers, multi-faceted and flexible mobilization strategies, and a convenient, welcoming vaccine clinic site [44]. Together, the successes and positive feedback we and others have experienced from using participatory planning with im/migrant groups to address COVID-19 challenges suggests that listening, engaging, and partnering with communities experiencing health disparities is an essential part of successful public health programming.

Additionally, our work as a community-academic partnership developing and implementing the CERC intervention also highlights the assets that im/migrants bring to COVID-19 education and vaccine efforts. Not only did im/migrant community partners create, translate, culturally adapt, and disseminate COVID-19 messaging, but they also organized clinics that facilitated vaccine access to members of their communities. This finding is in line with a study of refugee-led organizations in Kenya and Uganda. Betts, Easton-Calabria, and Pincock found five areas where refugees are or could respond to COVID-19 or other pandemics including: providing public information, filling capacity gaps, delivering healthcare, shaping social norms, and tracking disease [45].

RHCP’s ability to quickly pivot to COVID-19-related health inequities at the beginning of the pandemic also highlights how community engaged research partnerships are uniquely poised to address emerging public health issues. These partnerships ground their work in established trusting relationships, center the voices of historically marginalized populations, and use feasible, acceptable, and sustainable approaches. As a result, they can (1) use Rothman’s participatory planning [29] to co-create needed interventions that build on community assets and (2) leverage community-based research and evaluation infrastructure honed through past research experience [46]. Going forward, long-standing CBPR partnerships should be considered strategic allies in addressing pandemic-related health disparities.

### Limitations

It is important to note the limitations of this work including the small sample size. We recruited a subset of social network members from four CLs who were willing to participate in our evaluation. The size of the study population does not represent the depth of the four participating social networks. Additionally, we did not measure actual vaccine uptake in response to the CERC intervention or whether vaccine recipients identified as im/migrants which limits our conclusions about intervention impact. Finally, we did not ask whether participants had a primary care provider. Thus, it is possible that the large proportion of respondents (70%) who reported that they did not know whether a healthcare provider had recommended that they receive a COVID-19 vaccine was because they did not have a primary care provider. CBPR partnerships are highly contextual and thus our findings may be not generalizable to other settings. Future research should include qualitative data collection on how to adopt evidence-based crisis communication interventions in collaboration with specific populations.

### Policy implications

As public health practitioners, it is our responsibility to ensure that all people, including im/migrant populations, have access to essential healthcare resources such as accurate COVID-19 information and vaccines. This is not charity but rather equity work [47]. To appropriately allocate resources, it is critical that public health professionals, healthcare administrators, and policy makers (1) recognize im/migrant communities as a distinct group disproportionately impacted by COVID-19 [48], and (2) develop tailored interventions—such as the Crisis and Emergency Risk Communication (CERC) intervention and vaccine clinics described in this manuscript—in collaboration with those most impacted [49]. We encourage others to incorporate bidirectional communication into public health policies. Specifically, we recommend frameworks that leverage the expertise of community partners in leading messaging campaigns (rather than acting as conduits for dissemination). An important next step would be to document lessons learned, successful strategies, and resources developed from COVID-19 to mitigate inequities im/migrants may experience in future pandemics [50]. By partnering as equals, we can learn more about and together eliminate the disparities experienced by im/migrants.

## Conclusions

In this manuscript, we described the implementation of a bidirectional CERC intervention to promote vaccination equity as well as a community-engaged and community-based vaccine clinics. These promising interventions appear to positively affect vaccine uptake among im/migrant communities in Olmsted County. We encourage other researchers to use an assets-based approach in collaborating with im/migrant communities to further investigate the role of tailored messaging campaigns and vaccine clinics to address health disparities experienced by this group.

### Supplementary Information

**Additional file 1:** Example COVID-19 Messages.

## Data Availability

The datasets analyzed during the current study are available from the corresponding author on reasonable request.

## Abbreviations

CBPR: Community-based participatory research
CDC: Centers for Disease Control and Prevention
CERC: Crisis and emergency risk communication
COVID-19: Coronavirus disease 2019
MN: Minnesota
RHCP: Rochester Healthy Community Partnership
SD: Standard deviation
US: United States
