# Supplementary figures and images for: Addressing COVID-19 inequities using bidirectional crisis and emergency risk communication and vaccine clinic interventions: a descriptive study
Source: BMC Public Health. 2023 Aug 10;23:1517. doi: 10.1186/s12889-023-16410-3 (PMC10413496; doi:10.1186/s12889-023-16410-3)

**Additional File 1: Example COVID-19 Messages**

English


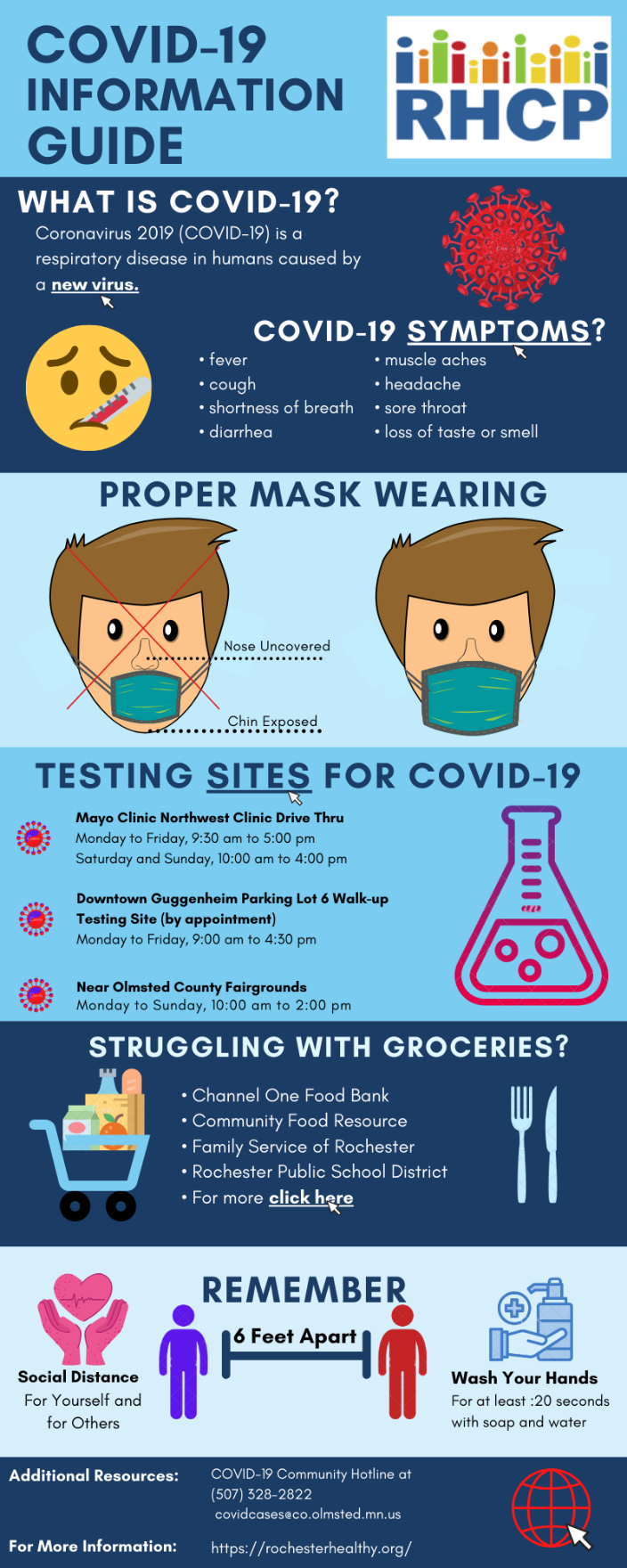


Khmer


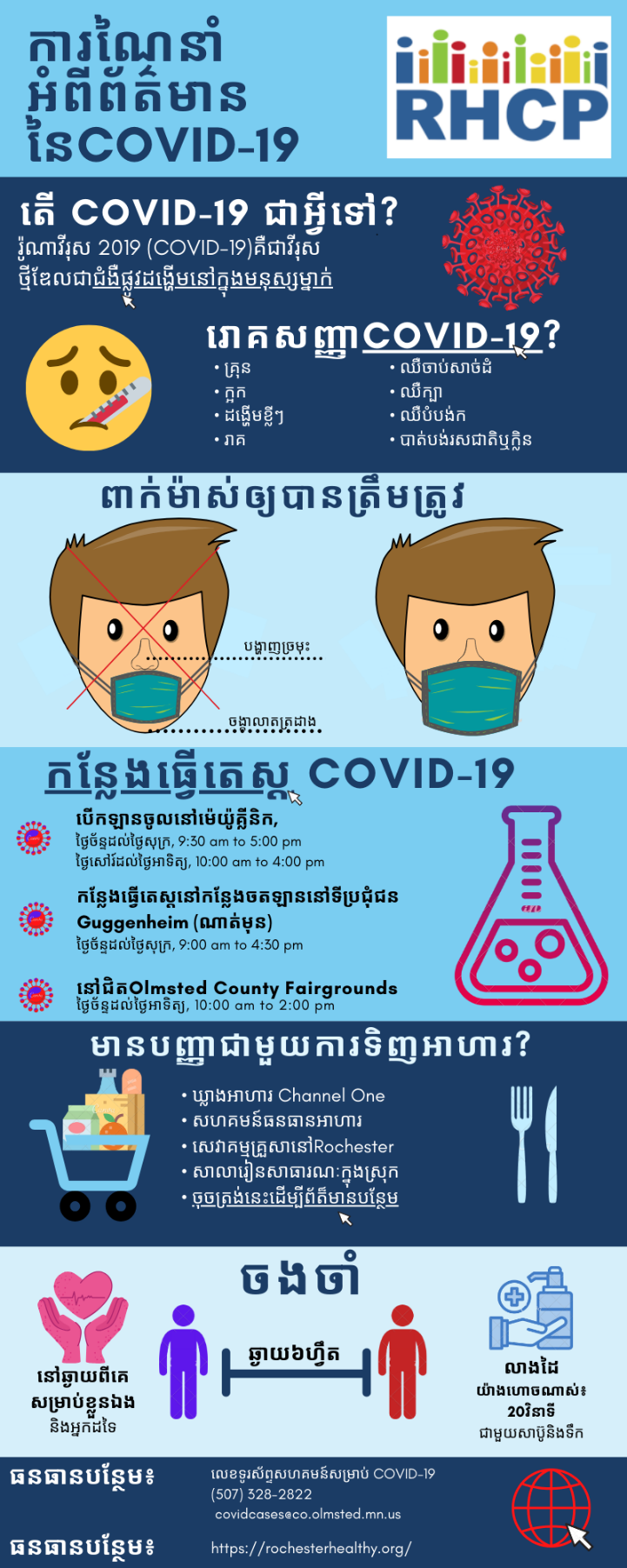


Spanish


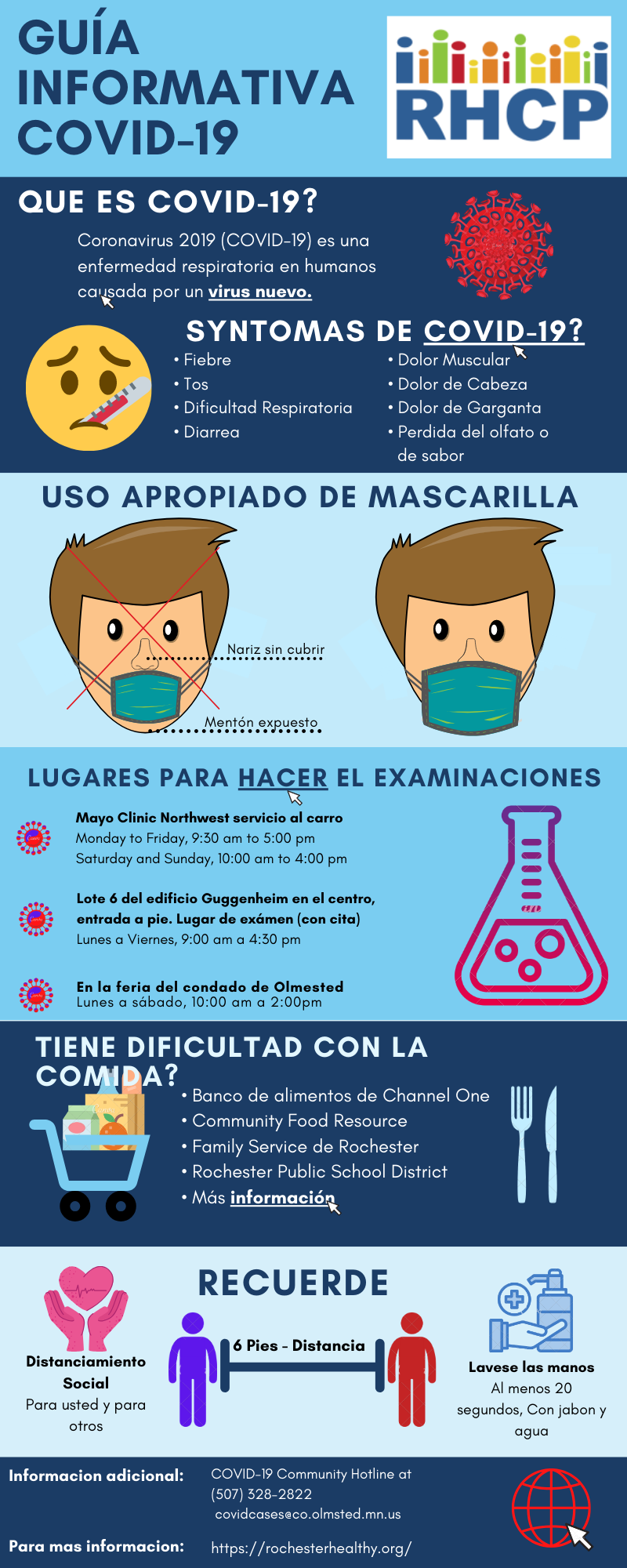

Supplement: Supplementary file 1 — Additional file 1: Example COVID-19 Messages. [file 12889_2023_16410_MOESM1_ESM.docx]
